# Analysis of perioperative fertility-related stress and associated factors in women of childbearing age undergoing salpingectomy for ectopic pregnancy: a study based on the health ecology model

**DOI:** 10.3389/fpsyt.2025.1637467

**Published:** 2025-12-04

**Authors:** Li Li, Zhen Guo, Ya-Li Guo, Jing-Ping Li

**Affiliations:** Department of Gynecology, Shanxi Provincial People’s Hospital, Taiyuan, Shanxi, China

**Keywords:** ectopic pregnancy, fertility-related stress, health ecology model, correlative factors, nursing, salpingectomy

## Abstract

**Objective:**

The objective of this study is to evaluate perioperative fertility-related stress and identify its correlative factors among women of childbearing age undergoing salpingectomy for ectopic pregnancy (EP).

**Methods:**

This retrospective cross-sectional study involved women of childbearing age who underwent unilateral salpingectomy (on the affected side) for EP in the Department of Obstetrics and Gynecology at a tertiary hospital in Taiyuan, Shanxi Province, China, between March 2023 and September 2024. Sociodemographic information and clinical data were collected from all participants. Additionally, participants completed two validated questionnaires: the Fertility Problem Inventory (FPI), which assesses fertility-related stress, and the Perceived Social Support Scale. Statistical analysis was performed using SPSS 23.0 software to identify factors associating fertility-related stress.

**Results:**

A total of 139 eligible women were included in the study. The mean fertility-related stress score of the patients was (148.39 ± 24.44), indicating high fertility-related stress. Factors associated with fertility-related stress covered all aspects of the health ecology model, including clinical and demographic characteristics (age, presence of lesions in the contralateral fallopian tube, and history of assisted reproductive technology use), psychological and behavioral characteristics (fertility intentions), interpersonal networks (perceived social support and number of children), working and living conditions (family monthly income per capita), and policy environment (degree of influence of traditional family fertility views).

**Conclusion:**

Women of childbearing age undergoing salpingectomy for EP experience high perioperative fertility-related stress. Our findings indicate that this stress is associated with factors across multiple levels, including clinical (e.g., older age, contralateral tubal lesions, history of ART), psychosocial (e.g., strong fertility intention, lower social support, having fewer children), and socioeconomic factors (e.g., lower income). These findings can guide healthcare providers in developing targeted interventions. Specifically, providers should not only focus on treatment outcomes but also address future fertility needs by assessing these key factors and leveraging social support networks to mitigate stress.

## 1 Introduction

Ectopic pregnancy (EP), also referred to as “extrauterine pregnancy,” is a common gynecological emergency characterized by the implantation and development of a fertilized egg outside the endometrial cavity of the uterus (1). EP is a prevalent condition among women of childbearing age, accounting for approximately 2% of all reported pregnancies (2–4). The incidence of EP has been steadily rising over the past 30 years. Although advancements in clinical management have reduced the associated mortality rate, EP remains the leading cause of maternal deaths during the first trimester of pregnancy (5). In China, since the implementation of the universal two-child policy, fertility levels have been steadily increasing, with a gradual optimization of the population structure and early signs of improvement in population aging. However, these changes have also led to a rise in the incidence of EP among women of childbearing age (6), particularly among those aged 35 and older, reversing previous trends (7).

It is estimated that approximately 98% of all reported EP cases are tubal pregnancies (8). More than half of these patients present with acute abdominal symptoms at the time of diagnosis, requiring emergency surgical intervention (9). Considering the preferences of patients for minimally invasive procedures, minimal scarring, lower economic costs, shorter hospital stays, faster postoperative recovery, and fewer postoperative complications (6, 7, 10, 11), laparoscopic salpingectomy on the affected side has emerged as the preferred surgical option for both clinicians and patients (3, 12).

While preserving the fallopian tube enhances future fertility potential, unilateral salpingectomy considerably reduces this possibility. Following laparoscopic salpingectomy on the affected side, only one fallopian tube remains available for future conception, thereby reducing the patient’s overall fertility potential (5). Boychuk et al. (13) found that the risk of infertility following unilateral salpingectomy is as high as 50%–60%. Consequently, this procedure can lower the likelihood of future intrauterine pregnancies in patients with EP, posing potential fertility risks. For women of childbearing age who have not yet achieved their desired family size or aspire future pregnancies, concerns over diminished fertility potential can result in considerable fertility-related stress or anxiety. Fertility-related stress is a multifaceted construct encompassing emotional distress, social pressure, marital strain, and concerns about one’s identity and future stemming from impaired fertility (14). Women undergoing salpingectomy for EP represent a uniquely vulnerable population. Unlike the general infertile population, they face the acute psychological impact of a pregnancy loss coupled with a surgical procedure that directly compromises their reproductive anatomy and potential. Recent evidence suggests that this iatrogenic diminishment of fertility can trigger significant grief, anxiety about future conception, and a heightened sense of reproductive uncertainty (15). These emotional states not only affect patients’ treatment decisions but may also adversely impact the clinical progression of EP. However, a comprehensive understanding of the factors associated with this specific stress in the perioperative context—particularly within a holistic framework that considers broader ecological influences—remains limited. Given these stressors, it is essential to explore the factors related to perioperative fertility-related stress in patients of childbearing age with EP undergoing salpingectomy. Such an investigation can offer valuable insights to support patients in making informed surgical decisions. It also highlights the need for healthcare providers to strengthen psychological care and support for such patients, as well as provide comprehensive fertility-related information during the perioperative period. Addressing these concerns proactively can alleviate the heightened fertility-related stress experienced by this vulnerable group and improve their overall well-being.

Health ecology model (HEM) provides a lens to review factors that contribute to this outcome. The model posits that individual health is the result of the interplay between individual factors and external environmental influences (16). It emphasizes the multi-layered influence of the social environment on individuals and the complexity of influencing factors. Health ecology model proposes five layers of influence on the health of an individual, personal traits, psychological and behavioral characteristics, interpersonal network, living and work conditions, external environmental factors such as society, economy, culture, and policies et al. (17) Health ecology model as a theoretical model is acknowledged as a useful framework applied for the research for individual health conditions and it has been widely applied in the management of healthy behaviors for chronic diseases, reproductive health prevention for eligible women and reproductive concerns of special populations (18–20).

In this study, the perioperative fertility-related stress in patients of childbearing age undergoing salpingectomy for EP was investigated, and the HEM was employed to identify factors potentially relating to this stress. Specifically, variables across all five levels of the HEM were examined, including clinical and demographic characteristics (e.g., age, tubal status, ART history), psychological and behavioral factors (e.g., fertility intention), interpersonal networks (e.g., social support, number of children), living and working conditions (e.g., income), and policy-related factors (e.g., influence of traditional fertility values). The aim of the study was to systematically screen for risk factors, improve health education strategies for perioperative patients with EP, and implement targeted perioperative interventions to reduce patients’ fertility-related stress during the perioperative period.

## 2 Participants and methods

### 2.1 Study participants

Women of childbearing age diagnosed with EP, who were admitted to the gynecology ward of a tertiary hospital in Taiyuan, Shanxi Province, China, between March 2023 and September 2024, were selected as the study subjects in this retrospective cross-sectional study.

The inclusion criteria were as follows: (1) women presenting with amenorrhea, abdominal pain, and vaginal bleeding, with positive blood or urine human chorionic gonadotropin (HCG) results, and transvaginal ultrasound confirming tubal pregnancy (21), who were scheduled to undergo unilateral salpingectomy; (2) aged 18–45 years; (3) with adequate communication, reading, and comprehension abilities; and (4) who provided informed consent along with their families.

The exclusion criteria were: women with (1) a history of psychiatric illness; (2) severe systemic diseases such as those affecting the heart, kidney, liver, or lung diseases, or malignancies that could impede cooperation; and (3) a confirmed diagnosis of infertility.

The sample size calculation was performed using PASS 2021 software. Based on an effect size (f²) of 0.25, a significance level (α) of 0.05, and a statistical power of 0.90 for the 17 independent variables included in the study, the minimum required sample size was determined to be 117 participants. To accommodate potential non-response or invalid responses, an additional 10% was included, resulting in a total of 139 individuals recruited. After excluding 11 ineligible cases, a valid sample was secured for analysis. The research strictly adhered to the principles outlined in the *Declaration of Helsinki*, ensuring data anonymity and ethical conduct. The study was approved by the Ethics Committee of Shanxi Provincial People’s Hospital, with approval number No (2024).802.

### 2.2 Survey content and tools

#### 2.2.1 Variable selection

The HEM categorizes the factors influencing individual health into five hierarchical levels: the first level is the core level, referring to the individual within the social-ecological environment, emphasizing the innate characteristics of the individual and the biological attributes of the disease; the second level is the psychological and behavioral level which pertains to the individual’s psychological traits and behavioral tendencies; the third is the interpersonal relationships level, that includes family and social networks; the fourth level includes living and working conditions that relate to the individual’s environmental and socioeconomic circumstances; and the fifth level consists of broader social, economic, cultural, and policy-related influences (17).

**Figure 1:**
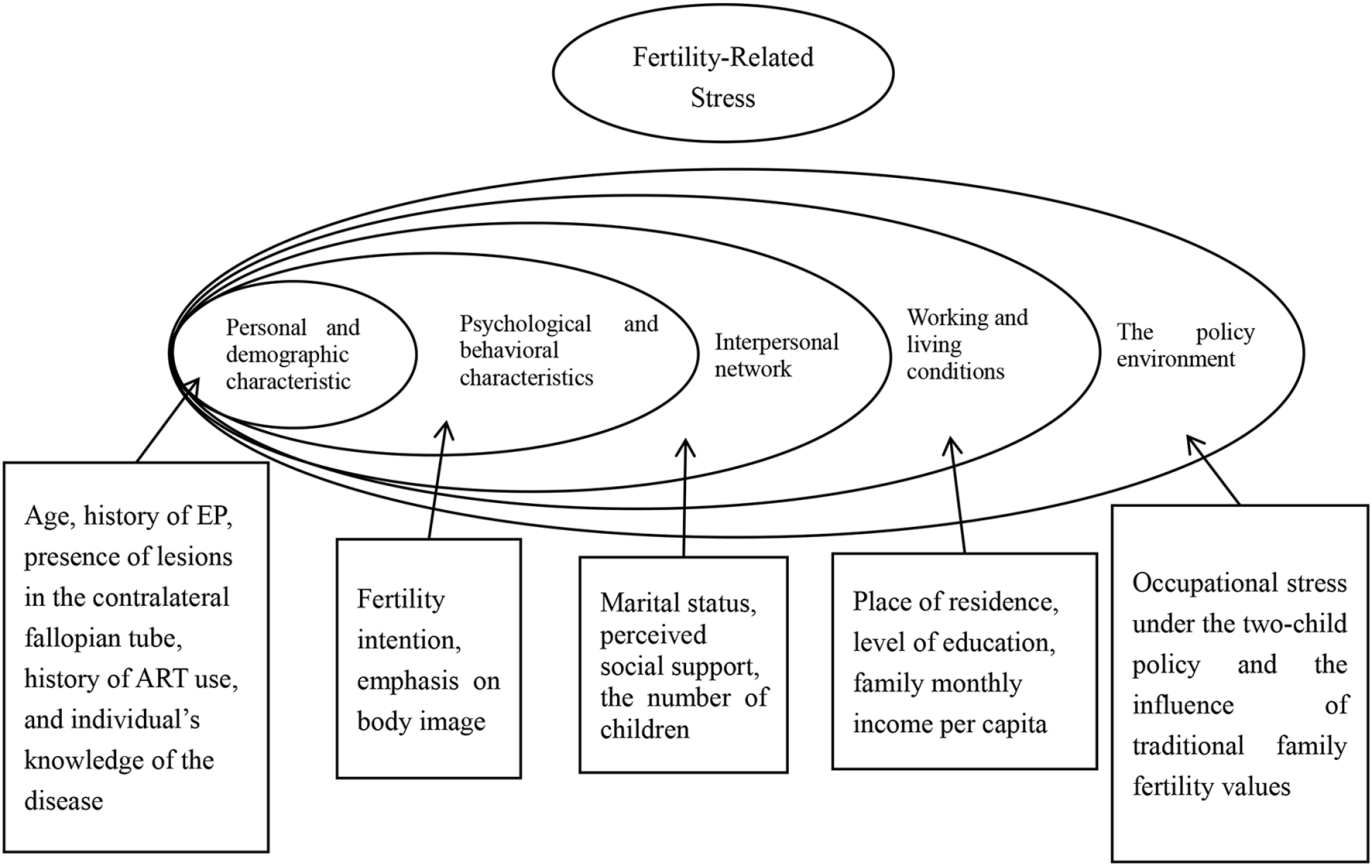
Hypothetical model of factors relating to perioperative fertility-related stress in patients of childbearing age undergoing salpingectomy for EP. Personal traits: Includes Age, history of EP, presence of lesions in the contralateral fallopian tube, history of ART use, and individual’s knowledge of the disease. Psychological and behavioral characteristics: Includes fertility intention and emphasis on body image. Interpersonal network: Includes marital status, perceived social support, and the number of children. Living and work conditions: Includes place of residence, level of education, and family monthly income per capita. Policy environment: Includes occupational stress under the two-child policy and the influence of traditional family fertility values.

Based on these five levels and a comprehensive literature review, the representative factors for each dimension of the HEM were preliminarily identified for this study. These include: (1) clinical and demographic characteristics, such as age, history of EP, individual knowledge of the disease, presence of lesions in the contralateral fallopian tube, history of assisted reproductive technology (ART) use, presence of chronic diseases, history of other gynecological or reproductive system diseases or surgeries; (2) psychological and behavioral characteristics, such as fertility intentions and the individual’s concern for body image; (3) interpersonal networks, including marital status, social support, and number of children; (4) living and working conditions, including place of residence, educational attainment, and family monthly income per capita; and (5) policy environment, including career pressure under the two-child policy and the influence of traditional views of the family about fertility. More details are presented in **Figure 1**.

#### 2.2.2 General information questionnaire

A self-designed questionnaire was used to collect sociodemographic and clinical information such as age, marital status, family monthly income per capita, level of education, number of children, and fertility intentions. Additionally, disease-related information, such as history of EP, presence of lesions in the contralateral fallopian tube, and presence of chronic diseases, was also recorded.

#### 2.2.3 Fertility problem inventory

The Fertility Problem Inventory (FPI) was developed by Newton et al. in 1999 (22) and was translated into Mandarin and adapted for Chinese populations by Peng et al. in 2011 (23) for assessing fertility-related stress in patients with infertility issues. The Chinese version of the FPI includes 46 items across 5 dimensions, which are social concerns, relationship concerns, need for parenthood, rejection of a childfree lifestyle, and sexual concerns. The questionnaire uses a 6-point Likert scale, with responses ranging from “strongly disagree” to “strongly agree.” Positive statements are scored directly (from 1 to 6 points), while negative statements are reverse-scored (from 6 to 1 point). The total score ranges from 46 to 276 points, with higher scores indicating greater fertility-related stress. The questionnaire has been widely validated for use in the assessment of fertility-related stress among patients with fertility problems and has been shown to have good psychometric properties (23). In this study, the Cronbach’s α coefficient for the questionnaire was 0.826, indicating good internal consistency.

#### 2.2.4 Perceived social support scale

The Perceived Social Support Scale (PSSS) was developed by Zimet et al. in 1988 (24) and was translated and adapted by Huang et al. (25) to measure the degree of perceived support from family, friends, and others. The scale consists of 12 items across 3 dimensions: family support, friend support, and other support. It uses a 7-point Likert scale, with scores ranging from 1 (strongly disagree) to 7 (strongly agree). The total score ranges from 12 to 84 points, with higher scores indicating higher levels of perceived social support. The scale has demonstrated strong reliability and validity. In this study, the Cronbach’s α coefficient for the scale was 0.910, indicating excellent internal consistency.

### 2.3 Quality control and data collection

#### 2.3.1 Pre-survey phase

A pre-survey was conducted with a sample of 10 patients to test the feasibility and clarity of the survey process. Based on feedback from the pre-survey, communication skills for data collection were refined and the inclusion of correlative factors was revised. The results of the pre-survey were consistent with the correlative factors that were initially selected, confirming their appropriateness for the main study.

#### 2.3.2 Survey phase

Before commencing the survey, investigators underwent centralized training to ensure consistency in administering the survey, and standardized instructions were used to explain the purpose, significance, and questionnaire completion methods to the participants.

#### 2.3.3 Data collection

The survey was conducted during non-treatment periods, with participants completing paper questionnaires on-site to collect data on fertility-related stress and correlative factors. For patients who had been discharged post-surgery, data was collected via telephone or email.

#### 2.3.4 Validation

Completed questionnaires were reviewed immediately on-site upon collection or during the telephonic call. For email responses, investigators checked the data within two days. If missing or invalid data was found, the investigator immediately contacted the participant for the missing details and ensured the information was complete. A total of 150 questionnaires were distributed: 123 were collected on-site, 5 via telephone, and 22 via email. During validation, 11 questionnaires (all from the email group) were identified as invalid due to extensive missing entries or patterned responses (e.g., selecting the same option for a majority of items). Subsequent attempts to contact these 11 participants for data verification or completion were unsuccessful (e.g., refusal to correct, unreturned calls/emails). These questionnaires were excluded from analysis, resulting in an attrition rate of 7.3% (11/150). No imputation was performed for missing data; only complete datasets were analyzed. Finally, 139 valid questionnaires were collected, with an effective response rate of 92.7%.

#### 2.3.5 Data entry phase

After the questionnaires were collected, two individuals independently entered the data into an Excel spreadsheet. Before and after importing the data into the SPSS statistical software, the data was double-checked by two people to ensure accuracy.

### 2.4 Statistical analysis

SPSS 23.0 statistical software was used for data analysis and processing.

Quantitative variables with a normal distribution were presented as mean (M) and standard deviation (SD), while qualitative variables were summarized as number (N) and percentage (%). Independent t-tests and ANOVA were conducted to examine differences in fertility-related stress across demographic and clinical characteristics. Correlations between fertility-related stress and perceived social support were evaluated using Pearson correlation analysis. To identify factors associated with fertility-related stress, multiple linear regression was performed. All tests were two-tailed, and a p-value< 0.05 was considered statistically significant.

## 3 Results

### 3.1 The Kolmogorov-Smirnov single-sample test

The K-S test demonstrated that both the total PSSS score and the total FPI score conformed to the criteria for a normal distribution (PSSS, *p* = 0.20; total FPI score, *p* = 0.089). Consequently, parametric testing methods were used to analyze the factors related to FPI scores. The mean fertility-related stress score of the patients was (148.39 ± 24.44), indicating high fertility-related stress (**Table 1**). A total of 139 participants were included in this study. The patients ranged in age from 18 to 45 years, and more than half were aged between 18 and 34 (57.55%). Most (77.70%) were without history of EP and chronic diseases. Regarding to the fertility intention, 56.83% of participants have fertility desires. About a quarter had received ART treatment (21.58%). About half having an educational level of college or above(45.32%), followed by emphasis on body image averagely(44.60%), resided in urban (43.17%) and family monthly income per capita (RMB) between 1,000 and 3,000 (41.73%). Detailed information is presented in **Table 2**.

**Table 1:** The total score of fertility-related stress and the scores of each dimension in patients of childbearing age undergoing salpingectomy for EP.

Item	Score range (points)	Average score (points, $\bar{x}\pm s$)
Global stress	46~276	148.39 ± 24.44
Social concerns	10~60	30.39 ± 5.86
Relationship concerns	10~60	30.28 ± 8.03
Need for parenthood	10~60	36.74 ± 10.01
Rejection of a childfree lifestyle	8~48	26.05 ± 8.43
Sexual concerns	8~48	22.91 ± 6.04

**Table 2:** Univariate analysis of perioperative fertility-related stress in patients of childbearing age undergoing salpingectomy for EP (n = 139, points, $\bar{x}\pm s$).

Variables	Cases [n (%)]	Fertility stress score	t/*F* value	*P*
Age				
18-34	80 (57.55%)	142.90 ± 23.61	-3.183	0.002
35-45	59 (42.45%)	155.83 ± 23.75		
Marital status				
Unmarried	16 (11.51%)	148.88 ± 19.73	0.094	0.910
Married	117 (84.17%)	148.11 ± 25.26		
Divorced	6 (4.32%)	152.50 ± 22.31		
Educational level				
Junior high school or below	22 (15.83%)	150.18 ± 23.81	2.178	0.117
High school or vocational school	54 (38.85%)	153.02 ± 24.78		
College or above	63 (45.32%)	143.79 ± 23.90		
Place of residence				
Urban	79 (56.83%)	145.49 ± 25.46	-1.611	0.109
Rural	60 (43.17%)	152.20 ± 22.69		
Chronic diseases				
Yes	31 (22.30%)	156.97 ± 24.09	-2.249	0.026
No	108 (77.70%)	145.93 ± 24.09		
Family monthly income per capita (RMB)				
<1000	28 (20.14%)	160.36 ± 20.94	3.399	0.020
1000~3000	58 (41.73%)	147.88 ± 24.15		
3000~5000	38 (27.34%)	142.76 ± 21.18		
≧5000	15 (10.79%)	142.27 ± 32.71		
Number of children				
0	51 (36.69%)	165.59 ± 16.77	45.013	<0.001
1 child	46 (33.09%)	148.09 ± 21.74		
2 or more children	42 (30.22%)	127.83 ± 18.66		
Fertility intention				
No	60 (43.17%)	130.10 ± 19.28	-10.131	<0.001
Yes	79 (56.83%)	162.28 ± 17.98		
Knowledge of disease				
Little/very little knowledge	55 (39.57%)	152.45 ± 25.11	1.773	0.155
Average knowledge	36 (25.90%)	150.64 ± 22.29		
Well-informed	34 (24.46%)	142.97 ± 23.32		
Very well-informed	14 (10.07%)	139.79 ± 27.63		
History of EP				
No	108 (77.70%)	145.09 ± 21.17	-2.477	0.018
Yes	31 (22.30%)	159.87 ± 31.22		
Presence of lesions in the contralateral fallopian tube				
No	83 (59.71%)	136.06 ± 20.41	-9.159	<0.001
Yes	56 (40.29%)	166.66 ± 17.56		
Other gynecological or reproductive diseases/surgical history				
No	98 (70.50%)	146.05 ± 23.89	-1.756	0.081
Yes	41 (29.50%)	153.98 ± 25.14		
Emphasis on body image				
Little/none	33 (23.74%)	148.15 ± 22.77	0.106	0.956
Average	62 (44.60%)	149.52 ± 26.51		
Important	30 (21.58%)	147.50 ± 24.05		
Very important	14 (10.07%)	145.86 ± 21.50		
Occupational stress under the two-child policy				
No	104 (74.82%)	147.32 ± 22.96	-0.799	0.428
Yes	35 (25.18%)	151.57 ± 28.53		
History of ART use				
No	109 (78.42%)	140.83 ± 21.57	-12.338	<0.001
Yes	30 (21.58%)	175.87 ± 10.67		
Influence of traditional family fertility values				
Little/no correlation	22 (15.83%)	131.09 ± 16.64	15.196	<0.001
Average correlation	34 (24.46%)	137.09 ± 27.10		
Significant correlation	54 (38.85%)	153.31 ± 19.56		
Very significant correlation	29 (20.86%)	165.59 ± 20.11		

### 3.2 Univariate analysis of perioperative fertility-related stress in patients of childbearing age undergoing salpingectomy for EP

There were significant differences in the total fertility-related stress scores (*p*< 0.05) in relation to various characteristics, including age, presence of chronic diseases, family monthly income per capita, number of children, fertility intention, history of EP, presence of disease in the contralateral fallopian tube, history of ART use, and the influence of traditional family fertility concepts. Additional details are provided in **Table 2**.

### 3.3 Correlation between perioperative fertility-related stress and perceived social support

Perioperative fertility-related stress in patients of childbearing age undergoing salpingectomy for EP was found to be negatively correlated with perceived social support and its dimensions (all *p*< 0.05), as shown in **Table 3**.

**Table 3:** Correlation analysis of perioperative fertility-related stress and perceived social support in patients of childbearing age undergoing salpingectomy for EP.

Item	Score (points, $\bar{x}\pm s$)	Correlation with fertility-related stress scores	
		*r* value	*P* value
Perceived social support	44.64 ± 12.10	-0.304	<0.001
Family support dimension	18.53 ± 5.50	-0.194	0.022
Friend support dimension	13.09 ± 4.97	-0.230	0.007
Other support dimension	13.02 ± 4.52	-0.250	0.003

### 3.4 Multivariate analysis of factors associated with fertility-related stress

Variable Assignment: For the independent variables, continuous variables were analyzed in their original form; the value assignment for categorical variables is shown in **Table 4**.

**Table 4:** Value assignment for independent variables.

Independent variable	Assignment method
Age	18~34 = 1, 35~45 = 2
Chronic diseases	No = 0, Yes = 1
Family monthly income per capita	<1000 = 1,1000~3000 = 2,3000~5000 = 3, ≧5000 = 4
Number of children	None = 0, 1 child = 1, 2 or more children = 2
Fertility intention	No = 0, Yes = 1
History of EP	No = 0, Yes = 1
Presence of lesions in the contralateral fallopian tube	No = 0, Yes = 1
History of ART use	No = 0, Yes = 1
Perceived social support	Original value
Influence of traditional family fertility values	Little/no influence = 1, Average = 2, Significant = 3, Very significant = 4

#### 3.4.1 Model diagnostics

The Durbin-Watson (D-W) statistic was 2.187, indicating no autocorrelation. All variance inflation factor (VIF) values were< 3, confirming the absence of multicollinearity. ANOVA test results showed *p*< 0.001, indicating that at least one variable significantly predicted the dependent variable (FPI score), suggesting that the establishment of the regression model was significant.

#### 3.4.2 Multivariate linear regression findings

Multivariate linear regression was used to identify the predictive variables (correlative factors) of fertility-related stress. As per the regression analysis results shown in **Table 5**, age, family monthly income per capita, number of children, fertility intention, presence of disease in the contralateral fallopian tube, history of ART application, perceived social support, and the influence of traditional family fertility concepts were found to be correlative factors for perioperative fertility-related stress in patients undergoing salpingectomy for EP (all *p*< 0.05). These variables collectively explained 68.7% of the total variance in fertility-related stress.

**Table 5:** Multiple linear regression analysis of factors relating to perioperative fertility-related stress in patients of childbearing age undergoing salpingectomy for EP (n = 139).

Factor	*B*	*SE*	β	*t*	*P*
Constant	140.635	9.317	–	15.095	<0.001
Age	6.026	2.607	0.122	2.312	0.022
Family monthly income per capita	-3.435	1.550	-0.128	-2.215	0.029
Number of children	-4.995	2.409	-0.167	-2.073	0.040
Fertility intention	13.311	3.798	0.271	3.505	0.001
Presence of lesions in the contralateral fallopian tube	10.384	3.252	0.209	3.193	0.002
History of ART use	11.174	4.020	0.189	2.780	0.006
Perceived social support	-0.261	0.126	-0.129	-2.075	0.040
Influence of traditional family fertility values	2.864	1.387	0.115	2.066	0.041

For the overall model, *R*² = 0.710, adjusted *R*² = 0.687, *F* = 31.269, *P*< 0.001.

## 4 Discussion

### 4.1 Patients of childbearing age with EP undergoing salpingectomy reported high levels of perioperative fertility-related stress

In this study, the average perioperative fertility-related stress score among patients of childbearing age with EP undergoing salpingectomy on the affected side was (148.39 ± 24.44). This score, while slightly lower than the total fertility-related stress score among women with infertility reported by Awtani et al. (26) and Wang and Li (27), was notably higher than the fertility-related stress scores of married women without fertility challenges (137.1 ± 25.0 points) noted by Yang et al. (28) This discrepancy may be because participants in our study were women of childbearing age with EP who retained the potential for future fertility as long as the contralateral fallopian tube remained healthy. However, it is also important to note that undergoing salpingectomy also imposes considerable emotional and psychological stress on patients undergoing this procedure.

The elevated fertility-related stress in this population can be explained by several factors. As China’s population ages, young women face the dual responsibilities of caring for older adults family members as well as raising children. The relaxation of national fertility policies has brought women’s fertility issues into the spotlight, highlighting the social and personal pressures associated with reproductive health and decision-making. Women of childbearing age with EP represent a special group, as they are not only in a stage of life where they are raising children, but they also worry about the necessity of surgery, the risk of recurrence of EP after surgery, and the potential impairment of their fertility post-surgery—all these concerns may further exacerbate their distress, leading to elevated levels of perioperative fertility-related stress in these patients. It highlights the need for healthcare providers to strengthen psychological care and support for such patients.

As shown in **Table 1**, firstly, the dimension of need for parenthood accounted for the largest proportion of fertility-related stress among all dimensions, which was similar to previous study (29). We inferred that might be due to the traditional beliefs and culture difference of China. In China, fertilizing children is usually regarded as the responsibility and obligation of women, and the role in reproduction and continuation of a family has become so deeply ingrained (30). Although in modern society, women have other ways to realize their values, Chinese women still regard motherhood as the main role and a respected identity in their minds (31). For those women of childbearing age with EP undergoing salpingectomy, the impaired reproductive organs, the desire for having children, and the uncertainty about future reproductive treatments and outcomes all increase the psychological burden of the patients (32). Secondly, we notice that the scores for stress in the social concerns and relationship concerns dimensions are no significant difference. This is because the social circles and friend circles that both spouses are exposed to are mostly the same, and thus the stress they experience are also similar (33). Interestingly, rejection of childfree lifestyle in the study was not shown a higher level in terms of the score for fertility-related stress. This may be related to the participants enrolled in the study among them a considerable portion have already at least one child, so the stress they experience in this dimension is lower than that of the dimension mentioned above. Finally, in terms of sexual concerns, the traditional views insist on “sex” correlated with fertility ability, and believe that “sex” is aimed at having offspring (34). For patients with subfertility, sexual life is considerable incline to the requisition for child-bearing (33).

### 4.2 Perioperative fertility-related stress in patients of childbearing age with ep undergoing salpingectomy was associated with multiple factors across different levels

In this study, various factors related to perioperative fertility-related stress, distributed across the five dimensions of the HEM were identified. In the dimension of clinical and demographic characteristics, the correlative factors included age, the presence of lesions in the contralateral fallopian tube, and history of ART use. In the psychological and behavioral aspect, fertility intention was identified. In the interpersonal network level, the correlative factors included the number of children and perceived social support. In the dimension of working and living conditions, family monthly income per capita was identified. Lastly, in the policy and environmental factors, the degree of association of traditional family fertility concepts was identified. The following section provides a detailed analysis of the main correlative factors:

(1) Personal and demographic characteristics represent the core dimension of the HEM. In this study, it was found that age correlates with perioperative fertility-related stress in patients. Patients aged ≥ 35 years who were undergoing salpingectomy for EP had higher perioperative fertility-related stress scores compared to those under 35, consistent with previous research findings (27, 35). This is because fertility is closely related to age; as women age, ovarian function declines, fertility risks increase, and fertility capacity gradually decreases, leading to increased concerns about future reproductive potential.

In addition, the condition of the contralateral fallopian tube also emerged as a critical factor determining fertility-related stress. The health of the contralateral fallopian tube directly affects the likelihood of future conception in women with EP of childbearing age (36). A survey on fertility outcomes after EP treatment showed that the intrauterine pregnancy rate within two years post-treatment for patients who underwent salpingectomy was 67%; this was lower than the intrauterine pregnancy rate of 76% for patients who underwent salpingostomy or drug treatment (37). This highlights the adverse impact of salpingectomy on fertility outcomes.

For women of childbearing age whose contralateral fallopian tube is affected by a lesion, the possibility of natural conception becomes unlikely, necessitating their reliance on ART. This dependence imposes a substantial financial burden on the family and poses significant physical and emotional challenges for the patient. Consequently, patients in these circumstances experience heightened perioperative fertility-related stress. These findings underscore the critical need for targeted interventions to address the specific challenges faced by this patient population.

Another result of this study was that patients with a history of ART use undergoing salpingectomy for EP experienced greater fertility-related stress compared to those without a history of ART. The likely reasons include the long treatment cycles, high costs, and physical and emotional trauma associated with ART, which impose a heavy financial and psychological burden on patients (38). Moreover, the occurrence of EP in these patients indicates a failure of ART to achieve a successful conception; the subsequent salpingectomy further compromises their reproductive potential. A combination of ART-related challenges, the emotional toll of an EP diagnosis, and the potential for impaired fertility post-surgery can exacerbate emotional distress, complicate decision-making processes, and worsen fertility-related stress (39). In clinical practice, healthcare providers should prioritize early identification and assessment of fertility-related stress in this high-risk group of patients. For modifiable factors, targeted and systematic intervention plans should be developed, involving both partners to ensure comprehensive support. Additionally, providing detailed preoperative information to these patients about the disease, treatment options, and potential outcomes is crucial to improving their understanding and awareness. This approach can help alleviate anxiety, facilitate informed decision-making, and improve psychological resilience during the perioperative period.

(2) Psychological and behavioral characteristics—positioned just beyond the core of the HEM—play a critical role in fertility-related stress.

The results of this study show that fertility intentions are associated with fertility-related stress, with patients who have a desire to conceive experiencing higher levels of stress. This is consistent with the findings of Anandavadivelan et al. (40) and Zhou et al. (41) Patients with EP having fertility intentions often worry about whether undergoing surgery and removal of the affected fallopian tube will relate to their fertility function. This concern creates a psychological conflict between their strong desire for fertility and the potential risks involved. Furthermore, such patients often have a corresponding need for fertility-related information. Upon being diagnosed with EP, they hope to discuss fertility-related topics with their physicians, including the impact of treatment on fertility and fertility preservation measures.

However, in clinical practice, the primary focus is on disease management, often at the cost of not addressing fertility preservation. Healthcare providers may lack adequate skills in communication about and imparting education regarding fertility-related issues (42). This disconnect results in patient dissatisfaction with treatment decisions, as their needs for timely fertility preservation information and interventions often go unmet. This can lead to regret and psychological distress, further increasing their fertility-related stress.

To address these challenges, healthcare providers should categorize patients based on their fertility intentions, with a special focus on those who express a desire to conceive. Early and accurate assessment of fertility intentions can help healthcare professionals predict fertility risks and recommend appropriate fertility preservation techniques. Providing multidisciplinary, comprehensive support, including information and intervention strategies, can help patients navigate fertility-related decisions more effectively, minimize the impact of EP and its treatment on fertility, and reduce feelings of regret associated with unmet fertility needs. Ultimately, such approaches can lower fertility-related stress and improve the overall well-being of patients.

(3) The interpersonal network, located in the third layer of the HEM, is another aspect that correlate with fertility-related stress. The study results indicate a significant negative correlation between perceived social support of patients and their fertility-related stress. This finding is consistent with the results of Li (33). Evidence suggests that strong social support systems help individuals maintain a positive physical and psychological state, enhancing their ability to cope with fertility-related challenges (43). Conversely, inadequate social support can exacerbate fertility-related stress (44).

In this study, the total perceived social support score of participants was relatively low at (44.64 ± 12.10), corresponding to a high level of fertility-related stress. Undergoing salpingectomy compromises the fertility potential of women, often triggering adverse psychological reactions such as anxiety, sadness, and feelings of inferiority. These negative emotional states may inhibit patients from openly expressing their concerns and seeking support from their spouses, family, or friends, thus limiting their ability to effectively utilize available social resources (45). As a result, inadequate social support exacerbates the difficulty of adapting to stressful events, further amplifying fertility-related stress.

Notably, research also emphasizes that perioperative interventions by healthcare professionals, including proactively addressing patients’ concerns and providing targeted perioperative health education for EP patients undergoing laparoscopy, can not only reduce anxiety and depression (46, 47) but also help patients better recognize the value of social support. These interventions may encourage patients to open up about their fertility-related worries to family or friends, thereby enhancing their ability to utilize interpersonal support resources and further alleviating fertility-related stress.

In addition, in this study, family support was identified as the highest-scoring dimension of perceived social support for patients, highlighting the crucial role of the family as the primary source of social support in mitigating fertility-related stress. Therefore, healthcare professionals should actively encourage patients to enhance their self-disclosure skills, enabling them to effectively communicate their emotions and concerns. By fostering positive interactions with their spouses and relatives, patients can harness the supportive role of their interpersonal networks to develop healthier perceptions and reduce fertility-related stress. Interventions should focus on empowering patients to leverage family support while also integrating broader social resources to address the multifaceted challenges posed by fertility-related stress.

Correlation of Number of Children on Fertility-Related Stress: The results of this study also show that the number of children a patient has associate with fertility-related stress, with childless patients experiencing more severe fertility-related stress. This is consistent with the findings of Wang et al. (48) In traditional Chinese culture, the family, as the basic unit of society, is perceived as incomplete without children. Children are regarded as the essential bond that sustains family harmony and stability, highlighting their undeniable importance in the societal and familial structure. For patients who have not yet had children, especially those beyond the optimal reproductive age, the loss of one or both fallopian tubes following salpingectomy may lead to the traumatic prospect of never having children (49). This situation often leads to profound psychological distress, including feelings of gender-based inadequacy, self-denial, and self-defeat. Additionally, pressure from spouses, extended family, and societal expectations can contribute to higher fertility-related stress in these patients. Moreover, the relaxation of the two-child policy in China has intensified the desire for children among women of childbearing age. Studies show that many Chinese families consider two children to be the ideal family size (50). As a result, patients with EP who already have one child may still experience fertility-related stress when faced with EP and the associated treatment decisions. However, for families with two or more children, the desire for additional offspring is lower, resulting in lower levels of fertility-related stress. These findings suggest the importance of healthcare professionals carefully tailoring interventions to address the specific fertility-related concerns of childless women with EP. The fertility aspirations of female patients must be integrated into treatment planning from the outset, ensuring informed and cautious decision-making.

(4) Working and living conditions are in the fourth layer of the HEM. Cao (51) reported that the lower the family’s monthly income, the higher the patient’s fertility-related stress—a finding that is corroborated by this study. Women with EP undergoing salpingectomy face the dual burden of potential infertility in the future and the prohibitive costs of ART. For many families with limited financial resources, these economic pressures, coupled with the strong cultural and personal desire for children, increase fertility-related stress.

Therefore, medical staff should proactively address these challenges by providing clear and comprehensive explanations of fertility-related information, including the implications of EP and treatment options. Such communication can help to reduce the patient’s fertility-related stress and equip them to make informed decisions. Furthermore, from a policy perspective, the relaxation of national fertility policies presents an opportunity to address these economic barriers. Lowering the costs of fertility treatments and incorporating certain ART procedures into medical insurance schemes would fundamentally boost the fertility confidence of women of childbearing age, helping to support their reproductive aspirations.

(5) The policy environment is the outermost layer of the HEM. The results of this study show that the extent to which traditional family fertility values influence individuals is an important factor related to fertility-related stress. The American sociologist Ogburn’s theory of cultural lag posits that “different parts of culture evolve at different rates, with institutional culture lagging behind material culture, and ideological culture lagging even further behind institutional culture, thereby creating a cultural lag.” (52) In this context, China’s family planning policies represent an institutional cultural shift. However, after the introduction of the revised policies, the accompanying cultural ideas regarding fertility failed to keep pace, creating a cultural lag that affects policy implementation. China’s deeply rooted traditional fertility culture, characterized by the preference for “more children” and “having a son,” has been deeply ingrained in people’s minds over millennia. Although this was reshaped to some extent by family planning policies, the persistence of cultural inertia continues to influence societal attitudes toward fertility (53). Fertility decisions, therefore, are not just a personal matter for women but also a collective decision of the family (54). For women with EP undergoing salpingectomy, which impairs their fertility potential, this cultural context can intensify their fertility-related stress. In families strongly influenced by traditional fertility norms, the inability to fulfill cultural expectations of having multiple children, or specifically a son, may lead to profound emotional distress. Women in such circumstances may face gender-based criticism, diminished familial status, or even marital discord. In extreme cases, the loss of fertility could even lead to the breakdown of marriages.

In modern society, where social media and technological innovations continuously reshape material needs and lifestyles, it is essential to foster a parallel change in mindsets. Addressing the cultural lag in fertility attitudes requires a concerted effort to promote modern values that respect gender equality in fertility decisions.

Healthcare providers and policymakers should consider the implications of traditional fertility culture when developing support systems for EP patients. Integrating these approaches into public health strategies can help reduce fertility-related stress among women of childbearing age, ensuring that institutional policies are better aligned with societal attitudes.

It is important to note that the participants in this study were predominantly married and highly educated. While our analysis within this sample did not find a significant association between educational level and fertility-related stress (*p*=0.117), suggesting that the core distress of fertility impairment may be a universal concern in this patient population, this demographic profile may limit the direct generalizability of our findings. Women who are unmarried, less educated, or have lower socioeconomic status may face additional, unique stressors (e.g., greater financial barriers to ART, less social support) that were not fully captured here. Therefore, the overall levels of stress we report might not fully represent the experiences of these underserved subgroups. Future research should intentionally seek to include more diverse populations to validate and extend our findings.

## 5 Conclusion

In this study grounded in the HEM, factors related to perioperative fertility-related stress in women of reproductive age with EP were analyzed. The findings revealed that these women experienced relatively high fertility-related stress. From the HEM perspective, the identified correlative factors included age, fertility intention, family monthly income per capita, number of children, social support, presence of lesions in the contralateral fallopian tube, history of ART use, and the extent of influence from traditional family fertility values. These results suggest that clinical healthcare professionals should pay particular attention to patients with the following characteristics: women who are older, have lower family monthly income per capita, are childless or have only one child, have fertility intentions, have a lesion in the contralateral fallopian tube, have a history of ART, and are significantly associated with traditional family fertility values. Especially when performing salpingectomy, along with effective disease treatment, it is critical to address the future fertility needs of patients. In addition, healthcare providers should encourage patients to leverage the positive role of social support networks to reduce their perioperative fertility-related stress.

### 5.1 Study limitations and future directions

This study has several limitations that should be acknowledged. First, the relatively modest sample size, though meeting the minimum statistical requirement, may still limit the statistical power for more complex subgroup analyses and could affect the stability of the multivariate regression model. Coupled with its single-center design, the generalizability (external validity) of our findings may be constrained. The patient population and clinical practices might not be fully representative of those in other regions of China or in different levels of healthcare settings, potentially limiting the extrapolation of our conclusions. In especial, the generalizability of our findings is constrained by the sociodemographic characteristics of our sample, which was predominantly composed of married, highly educated women receiving care at a single tertiary hospital. This profile is likely influenced by selection bias (e.g., health awareness, access to care) and differential exposure risks for EP (e.g., older maternal age, ART use associated with higher education). Consequently, our results may be most representative of this specific subgroup and should be extrapolated with caution to unmarried, less-educated, or socioeconomically disadvantaged populations who may experience different challenges and levels of fertility-related stress. Future multicenter studies employing community-based or diverse sampling strategies are needed to confirm the universality of our results across a broader sociodemographic spectrum.

Second, the cross-sectional nature of this study design is an inherent limitation. It captures fertility-related stress and its associated factors at a single point in time, predominantly during the perioperative period. Consequently, it precludes any assessment of the trajectory of fertility-related stress over time (e.g., from diagnosis to long-term recovery) and cannot establish causal relationships between the identified factors and stress levels. The observed associations should be interpreted as correlational. Furthermore, while the study was based on the comprehensive HEM framework, the cross-sectional data cannot fully capture the dynamic interactions between the different levels of factors postulated by the model.

Third, although we minimized the time between the perioperative experience and data collection (on-site for inpatients, within 0–2 days of discharge for others) to reduce recall bias, the cross-sectional and retrospective assessment of stress remains a limitation. Furthermore, for women with a history of assisted reproductive technology (ART), the assessed perioperative stress, while intended to focus on the current ectopic pregnancy event, may be influenced by the emotional burden and prior experiences of infertility and ART treatment failures. Although we identified ART history as a significant correlate of increased stress, our study design cannot delineate the precise contribution of the past ART experience versus the current surgical event to the overall stress reported. This interplay warrants investigation in future longitudinal studies that measure stress at multiple time points, including before any fertility treatments begin.

Finally, although the HEM provided a valuable structural framework, our study primarily relied on quantitative methods and self-report measures. This approach may not fully capture the nuanced, subjective, and deeply personal experiences of fertility-related stress following salpingectomy. Factors such as complex emotional processing, the quality of patient-clinician communication, and subtle cultural dynamics within the family might be better explored through qualitative methods.

Based on these limitations and our findings, we propose the following directions for future research: Future research should employ prospective, multicenter designs with larger sample sizes. This would enhance the statistical power, allow for more robust subgroup analyses (e.g., stratifying by ART history or contralateral tubal status), and improve the generalizability of the findings across diverse socioeconomic and cultural contexts. Longitudinal studies are crucial to track the evolution of fertility-related stress from diagnosis through treatment and into long-term follow-up. Such designs can help establish temporal precedence, identify critical intervention time points, and better understand the causal pathways linking clinical and psychosocial factors to stress outcomes. To gain a deeper, more holistic understanding, future studies should integrate qualitative methodologies (e.g., in-depth interviews or focus groups) with quantitative surveys. This would help uncover the rich, lived experiences of these women, elucidate the mechanisms behind the statistical associations we found (e.g., how exactly traditional values or low income exacerbate stress), and identify potentially overlooked influencing factors. The significant factors identified in this study, such as low social support, strong fertility intention, and financial pressure, present clear targets for intervention. Future work should focus on developing and testing tailored psychosocial interventions, such as structured counseling programs, peer support groups, or couple-based therapies, aimed at mitigating these specific stressors. The effectiveness of these interventions should be evaluated through randomized controlled trials. In addition, future research could also include the partners of women undergoing salpingectomy to understand the dyadic impact of fertility stress and explore factors influencing support provision. Additionally, comparing fertility-related stress across different surgical approaches (e.g., salpingectomy *vs*. salpingostomy) could provide valuable insights for shared decision-making.

## Data Availability

The original contributions presented in the study are included in the article/supplementary material. Further inquiries can be directed to the corresponding author.
